# Reduction of Intramedullary Apoptosis after Stem Cell Transplantation in Black African Variant of Pediatric Sickle Cell Anemia

**DOI:** 10.4084/MJHID.2014.054

**Published:** 2014-07-07

**Authors:** Antonella Isgrò, Pietro Sodani, Marco Marziali, Javid Gaziev, Daniela Fraboni, Katia Paciaroni, Cristiano Gallucci, Gioia De Angelis, Cecilia Alfieri, Michela Ribersani, Daniele Armiento, Andrea Roveda, Marco Andreani, Manuela Testi, Guido Lucarelli

**Affiliations:** 1 International Center for Transplantation in Thalassemia and Sickle Cell Anemia, Mediterranean Institute of Hematology, Policlinic of the University of Roma Tor Vergata., Rome, Italy; 2 Laboratory of Oncohematology, Department of Laboratory Medicine, Policlinic of the University of Roma Tor Vergata, Rome, Italy

## Abstract

**Background and Purpose:**

Allogeneic hematopoietic stem cell transplantation (HSCT) is the only curative treatment for sickle cell anemia (SCA). We report our experience with transplantation in children with the Black African variant of SCA and the effects of transplant on erythroid compartment in bone marrow (BM).

**Patients and methods:**

Twenty-seven consecutive patients who underwent BM transplantation from HLA-identical donors following a myeloablative conditioning regimen were included. Using both CD71 and FSC parameters, we obtained three erythroid populations: EryA–C. Ery A (CD71^high^ FSC^high^) are basophilic; Ery B (CD71^high^ FSC^low^) are late basophilic and polychromatic; and Ery C (CD71^low^ FSC^low^) are orthochromatic erythroblasts and reticulocytes. To analyze the effect of transplantation on intramedullary apoptosis, we studied Fas (CD95+) and caspase-3 expression in erythroblast subpopulations.

**Results:**

All patients experienced sustained engraftment, and all surviving patients remained free of SCA-related events after transplantation. The erythroid population showed expansion in the BM at baseline. After transplant, levels decreased, especially of Ery C, in parallel to reduced Fas expression and an initial caspase 3 increase in erythroid population, similar to reported later steps of “normal” erythroid maturation.

**Conclusions:**

The results suggest a good chance of cure for children with SCA, with an excellent survival rate. We also observed “normalization” of erythroid populations in parallel with a decreased intramedullary apoptosis rate, suggesting normal erythroid maturation in ex-SCA patients after HSCT.

## Introduction

Sickle cell disease is a group of genetic conditions in which pathology results from the inheritance of the sickle cell gene variant either homozygously or as a double heterozygote with another interacting gene. The spectrum of resulting conditions is influenced by the geography of individual hemoglobin genes, but in most populations, the commonest genotype at birth is homozygous sickle cell (SS) disease. Since this genotype involves a greater mortality, the relative proportion of sickle cell genotypes is influenced by age as well as by the geographical distribution of individual genes.^1^

The sickle cell trait is widespread throughout Africa. Frequencies are low (<1%–2%) in the north and south of the continent but high with variable frequencies throughout much of equatorial Africa. SCA is characterized by a cascade of events that begin with the polymerization of hemoglobin S and the sickling of red blood cells. Following these events is occlusion of small and larger vessels because of the adherence of the sickled cells to the vascular endothelium, which leads to pain crises, stroke, acute chest syndrome, and multi-organ failure as the most frequent complications.^2^ In patients with the Black African variant, painful crises, chest syndrome, and stroke are more frequent and appear earlier in life. The causes of death are strongly influenced by the prevalence of malaria and other infections, and almost certainly by the availability and sophistication of medical and other services. In sub-Saharan Africa, survival is markedly shortened, and median survival may be as short as 5 years.

Hematopoietic stem cell transplantation (HSCT) is the only radical cure for this genetic disorder,^3^ and to date, several hundred patients have undergone gene-identical HSCT.^4^–^9^ In accordance with data recently published,^25^ our experiences confirm that it is possible to offer a good chance of cure to children with SCA, and we thus have made the following recommendation: “*HSCT should be considered the standard of care for SCA children with a human leukocyte antigen-identical donor, before complications result from the sickling of red blood cells*.”

Less well established is the potential contribution of ineffective erythropoiesis to the pathophysiology of this hemoglobinopathy. As in thalassemia patients, an expansion of erythroid precursors is observed in SCA patients at the bone marrow (BM) level but is less severe than in thalassemia.^10^–^11^ Normal homeostasis of the erythropoietic system requires an appropriate balance between the rate of erythroid cell production and red blood cell destruction. Growing evidence indicates that apoptotic mechanisms play a relevant role in the control of erythropoiesis under physiologic and pathologic conditions.^10^ Death receptors of the TNF receptor superfamilies (Fas-Ligand (Fas-L), TNF-α, TRAIL) activate the extrinsic apoptotic pathway. Fas and Fas-L are expressed in cultured erythroblasts, but there are controversies regarding the level and differentiation stage at which they are expressed. Some studies suggest the existence of a negative regulatory feedback operating at low erythropoietin (Epo) levels in a paracrine pathway. In this system, Fas-L–expressing mature erythroblasts display cytotoxicity against immature erythroblasts expressing Fas.^12^,^13^ Epo can partially protect immature erythroid cells from Fas-mediated apoptosis; thus, Fas and Fas-L are major regulators of erythropoiesis.

Both proteins are downregulated in BM or spleen in proerythroblast and basophilic cells in β-thalassemic mice compared to control mice in vivo. This downregulation of Fas/Fas-L expression might be a marker of erythropoietic stress and explain, at least in part, erythroid expansion in thalassemia.^14^

We hypothesized that Fas might contribute to the cell death of SS erythroid precursors at the BM level, but that transplant may be corrective. Here we report our experience with transplantation in a group of pediatric patients with Black African variant SCA, who received transplantations from HLA-identical siblings. We analyzed the effect of transplant on erythropoiesis and intramedullary apoptosis, studying Fas (CD95+) and caspase-3 expression in erythroblast subpopulations before and after transplant. We also used this opportunity to directly compare the differentiation and survival of SCA and donor (AA or AS trait carrier)-derived erythropoiesis in vivo.

## Patients and Methods

This study included 27 consecutive SCA patients who underwent BM transplantation from HLA-identical sibling donors between January 2010 and June 2013. Twenty-seven patients with the Black African SCA variant were treated with a modification of our Protocol 26, which was in use for Class 3 thalassemia patients^15^ (here identified as Protocol 28). The institutional review board approved the treatment protocol, and all parents of patients provided written informed consent in accordance with the Declaration of Helsinki.

### Patient characteristics

The median patient age was 10 years (range 2–17 years), and the median donor age was 11 years (range 1–26 years). Patient characteristics at the time of transplantation are summarized in Table 1. All patients showed good performance status (Lansky/Karnofsky 100) before transplantation. No patient had a splenectomy before transplantation, and only two received chronic blood transfusions; the serum ferritin level before transplantation was 278 ± 231 ng/mL (mean ± SD). Before transplantation, 11 patients had recurrent, painful, vaso-occlusive crisis; nine patients had recurrent painful crisis in association with acute chest syndrome; three patients experienced ischemic stroke and recurrent vaso-occlusive crisis; two patients experienced ischemic stroke; one patient exhibited leukocytosis, and one patient exhibited priapism.

HLA typing at the molecular level was performed, and all donors for both groups were fully matched.

### Transplantation procedure

Patients received fludarabine (30 mg/m^2^/day) for 5 days and a conditioning regimen including targeted intravenous busulfan (14 mg/kg total dose) and cyclophosphamide (200 mg/kg total dose). All patients received cyclosporine A, low-dose methylprednisolone, and a short course of methotrexate as GVHD prophylaxis. Among the patients, six had cyclosporine A-related neurotoxicity with seizures. All patients received valproic acid (Depakin; Sanofi-Aventis) at a dose of 30 mg/kg/day in 3 divided doses starting at 24 hours before the first busulfan administration. Many risk factors for the development of CSA-related neurotoxicity have been investigated in our patients, including arterial hypertension, fluid overload, hypercholesterolemia, hypomagnesaemia and pre-existing brain disease. In the screening examinations of these patients the brain magnetic resonance imaging (MRI) showed gliosis in 11/27 stroke free Black African SCA patients (manuscript in preparation). The brain MRI finding, usually associated to CSA neurotoxicity, was posterior reversible leukoencephalopathy syndrome (PRES), typically distributed in the posterior regions of the white matter of the brain. We cannot rule out the pre-existing brain disease in these patients might predispose to seizures during CSA treatment. In general, the prognosis of CSA neurotoxicity has been good and posterior leukoencephalopathy usually resolved completely with dose reduction or drug withdrawal. As alternative GVHD prophylaxis, we opted for tacrolimus. This calcineurin inhibitor, although similar to CSA in mechanism and metabolism, did not produce neurological side effects in these patients.

Children with Black African variant SCA were prone to invasive infections caused by S. pneumonia, H. influenzae and Plasmodium falciparum (in malarial areas). Malaria is more endemic in Black African areas and therefore malaria is more common in Black SCA patients. In Africa, malaria contributes substantially to the early mortality of patients with SCA. For these reasons we preferred in this population fludarabine-based preparative protocols, well tolerated, with less immunosuppression and minimal toxicity.

All patients received BM from HLA-identical sibling donors 36 h after the final dose of cyclophosphamide, and all donors with sickle cell trait received hyperhydration and blood transfusion before the multiple marrow aspirations. The median number of total nucleated cells infused was 4.08 × 10^8^/kg (range 1.7 × 10^8^/kg to 10.0 × 10^8^/kg), and the median number of CD34 cells was 5.8 × 10^8^/kg (range 1.2 × 10^6^/kg to 11.2 × 10^6^/kg).

The diagnosis and degree of acute and chronic GVHD were assessed according to standard criteria.^16^,^17^ All patients were given prophylactic broad-spectrum antibiotics and antifungal drugs until the neutrophil level exceeded 1.0 × 10^9^/L, and also received acyclovir as herpes virus prophylaxis and trimethoprim/sulfamethoxazole as *Pneumocystis jiroveci* prophylaxis. Patients were monitored weekly for the presence of Epstein-Barr virus, cytomegalovirus (CMV), adenovirus, and BK virus in the blood and/or urine using sensitive reverse transcriptase polymerase chain reaction (PCR), from the beginning of transplant preparation until at least 100 days post-transplant.

### Assessment of chimerism

The first chimerism analysis was performed on BM samples obtained 20 days after transplant to determine the percentage of donor/recipient DNA using PCR-based analysis of short tandem repeats. Subsequently, at 60, 90, 180, and 365 days post-transplant, lineage-specific chimerism analysis was performed by PCR using fluorescent primers flanking a single informative short tandem repeat (AmpFLSTR Profiler Plus; Applera, CA, USA) previously identified to be polymorphic between the patient and donor.

### Cytometric assay for erythroid cell precursors

We previously developed a flow cytometric assay to identify stage-specific erythroblasts directly in hematopoietic tissue (BM) based on their expression of the transferrin receptor (CD71), which declines with erythroblast maturation.^18^ However, the decline in CD71 appeared to be gradual, without the formation of well-resolved subpopulations. In this study, we distinguished well-resolved erythroblast subpopulations by considering, in addition to CD71, the forward scatter (FSC) parameter. FSC is a function of cell size and has been used previously to assess erythroblast maturation independently of cell surface marker expression. When the cells were analyzed using both CD71 and FSC parameters, they consistently resolved into three principal subpopulations, which we labeled Ery A, Ery B, and Ery C erythroblasts. Ery A (CD71^high^ FSC^high^) are basophilic; Ery B (CD71^high^ FSC^low^) are late basophilic and polychromatic; and Ery C (CD71^low^ FSC^low^) are orthochromatic erythroblasts and reticulocytes.^11^,^14^

Bone marrow specimens of patients and donors were obtained to evaluate Fas and caspase 3 expression in erythroblasts: anti-CD95 PE, anti-CD71 FITC, and anti-CD45 PercP Cy5.5 were mixed in a tube. A volume of 10 μL of these MoAb cocktails (BD, Becton Dickinson, San Diego, CA, USA) was combined with 100 μL of bone marrow mononuclear cells for 10 minutes at room temperature, then lysed with BD Pharm Lyse 1× for 20 minutes at room temperature and washed with 2% phosphate-buffered saline plus bovine serum albumin. Samples were analyzed with BD FACS Canto II and the software, BD FACSDiva.

### Statistical analysis

The probabilities of survival, SCA-free survival, rejection, and mortality were calculated using Kaplan–Meier curves.^19^

Non-parametric statistics was used *(Mann-Whitney, Wilcoxon test*) for unpaired and paired comparisons between the parameters analysed in patients and healthy individuals. A p-value less than 0.05 was considered significant. Statistical analyses were performed by using Stat View 5.0 software (SAS Institute, Cary, NC, USA).

## Results

### Clinical assessment post-transplant

The median time to neutrophil recovery (absolute neutrophil count ≥ 500 × 10^9^/L on 3 consecutive days) was 16 days (range 11– 23 days). Platelet recovery ≥20 ×10^9^/L was observed at a median of 17 days (range, 11–22 days) after allo-HSCT. In terms of platelet transfusion needs, the median number of platelet units transfused in the first 100 days after HSCT was 15 U (range, 0–53 U) but it increased when complications as severe acute GVHD or sepsis appeared. In our cohort of patients we observed a lower rate of platelet transfusion and faster platelet recovery kinetics after HSCT, but also highlighted the negative effect of severe acute GVHD as a risk factor for increased need for platelet transfusions.

At 2 months after transplantation 3 patients had donor chimerism between 95% and 98%, and all the remaining patients had full donor chimerism. At the last control, all patients experienced sustained engraftment with 100% donor chimerism. All patients and donors except one had positive serology for CMV before transplantation. Asymptomatic CMV reactivation occurred in 26 of 27 patients. All patients were provided pre-emptive antiviral therapy, and none developed CMV disease.

Seven patients developed grade 2 acute GVHD of the skin, and five patients developed grade 3–4 GVHD, principally after 30 days post transplant. All patients responded promptly to the steroid treatment administered to control acute GVHD (1–2 mg/kg/day prednisone). At present, all patients except one are off immunosuppressive medication. Chronic GVHD was observed in four patient: one patient developed bronchiolitis obliterans, and one patient had severe chronic GVHD with intestinal and hepatic involvement until death, as a result of multi-organ failure at day +190 post-transplantation. Cumulative incidence of grade 3–4 acute GVHD was 18%. Cumulative incidence of persistent severe chronic GVHD was 14%.

One patient died at 77 days post-transplantation from complications of severe GVHD of the gut. One patients died from multiorgan failure at 190 days post-transplantation. He had no steroid responsive grade 4 acute GVHD of the gut and developed sepsis, which led to multiorgan failure and death. One patient died from complications of bronchiolitis obliterans at 445 days post-transplantation.

After transplantation, no patients experienced complications typical of SCA, such as pain, stroke, or acute chest syndrome. The probabilities of survival, SCA-free survival, and transplant-related mortality after transplant were 89%, 89%, and 11%, respectively.

### Expression of Fas and caspase-3 on erythroid population at the BM level

**Figure 1:**
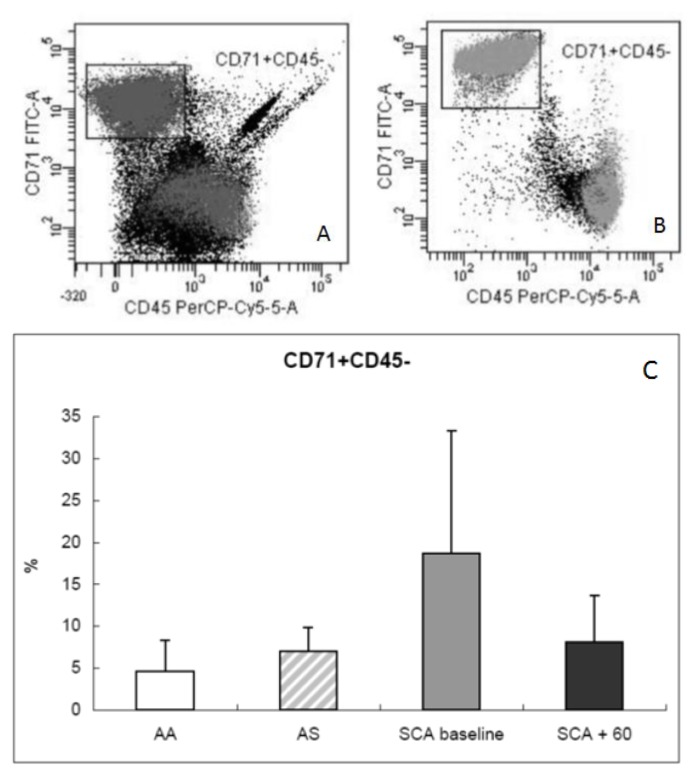
Flow cytometric analysis of bone marrow cells. (A, B) Bone marrow cells labelled with antibodies against CD71 and CD45. (A) Plot of CD71+CD45− versus FCS in SCA patients at baseline. (B) Plot of CD71+CD45− versus FCS in SCA patients at 60 days post-transplant (TMO). (C) Comparison of the percentages of erythroid precursors (CD71+CD45−) at the bone marrow level in normal donors (white bar), in AS trait carrier donors (dashed bar), in SCA patients at baseline (gray bar), and after 60 days post-transplant (black bar). The results are expressed as mean ± SD.

**Figure 2:**
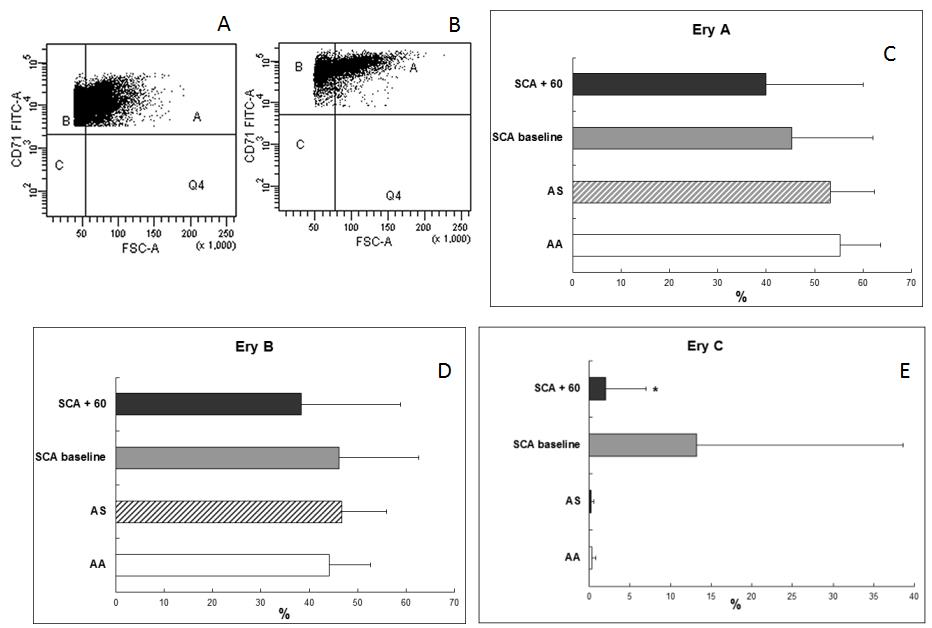
Flow cytometric analysis of erythroid subpopulations at the bone marrow level. (A, B) Primary bone marrow cells were simultaneously stained with CD71+CD45−. We distinguished erythroblast subpopulations (Ery A, Ery B, and Ery C) by considering, in addition to CD71, the forward scatter (FSC) parameter in the gated erythroid population. Ery A is CD71^high^ FSC^high^, Ery B is CD71^high^ FSC^low^, and Ery C is CD71^low^ FSC^low^. (A) Erythroid subpopulation in SCA patients at baseline. (B) Erythroid subpopulation in SCA patients 60 days post-transplant. (C–E) Comparison of percentages of Ery A, Ery B, and Ery C in SCA patients at baseline (gray bar), in SCA patients 60 days post-transplant (black bar) vs. normal donors (white bar) and AS trait carrier donors (dashed bar). The results are expressed as mean ± SD; * p= 0.0028.

**Figure 3:**
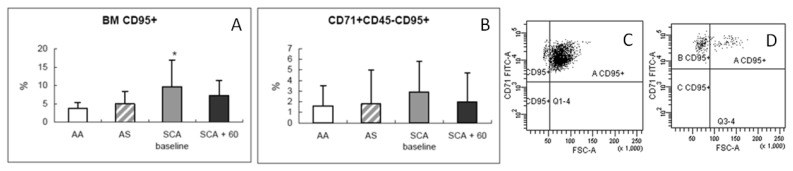
(A, B) Comparison of percentages (mean ± SD) of Fas expression at the bone marrow level (BM CD95+) and on erythroid populations (CD95+CD71+45−) in normal donors (white bar), in AS trait carrier donors (dashed bar), in SCA patients at baseline (gray bar), and after 60 days post-transplant (black bar). (C, D) Flow cytometric analysis of erythroid subpopulations (Ery A, Ery B, and Ery C) for CD95+ expression in SCA patients at baseline (C) and after 60 days post-transplant (D); * p= 0.004.

**Figure 4:**
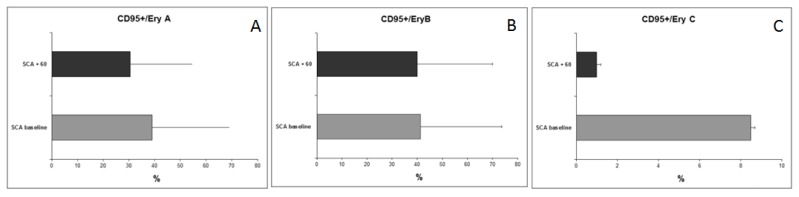
(A, B, C) Comparison of percentages (mean ± SD) of Fas expression on erythroid subpopulations (A: Fas expression on Ery A; B: Fas expression on Ery B; C: Fas expression on Ery C) in SCA patients at baseline (gray bar), and after 60 days post-transplant (black bar).

We observed an expansion of the BM erythroid population at baseline, probably as an essential process needed to maintain a constant red cell production in SCA patients (Fig. 1). Average percentages of CD71+CD45− were 4.6 ± 3.7% in normal AA donors, 7 ± 2.8% in AS trait carrier donors, 18.7 ± 14.6% in SCA patients at baseline, and 8.1 ± 5.6% in SCA patients at 60 days after transplant. After HSCT, decreased levels were observed in all three erythroid subpopulations (average 40 ± 20% *vs*. 45.3 ± 16.7% at baseline for Ery A; 38.4 ± 20.5% *vs*. 46.2 ± 16.4% at baseline for Ery B), especially for Ery C (2 ± 5% *vs*. 13.2 ± 25.4% at baseline; p= 0.0028) (Fig. 2) in parallel to a reduction in Fas expression (Fig. 3) in the BM (average CD95+CD34+, 3.7 ± 1.7% in normal AA donors; 4.9 ± 3.3% in AS trait carrier donors; 9.6 ± 7.3% in SCA patients at baseline, p= 0.004 *vs.* healthy controls; and 7.3 ± 4% at 60 days after transplant, p= 0.007 *vs*. healthy controls) and specifically in the erythroid compartment (average CD95+CD71+CD45−, 1.6 ± 1.9% in normal AA donors; 1.8 ± 3.2% in AS trait carrier donors; 2.9 ± 2.9% in SCA patients at baseline; and 2 ± 2.7% at 60 days after transplant) (Fig. 3). After transplant, a tendency to a normalization of erythropoiesis has been observed in our patients, with a reduction of Fas expression on three erythroid population but especially on more mature erythroid precursors Ery C (Fig. 4).

**Figure 5:**
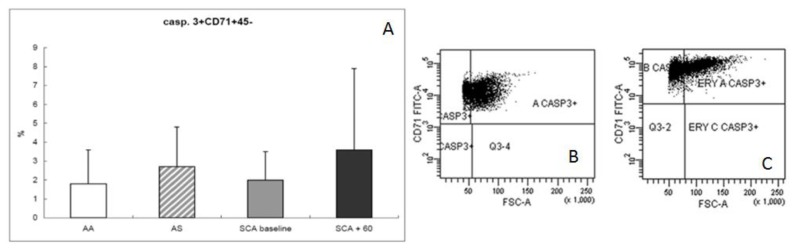
(A) Comparison of the percentages (mean ± SD) of erythroid precursors expressing caspase 3 (casp. 3+CD71+CD45−) at the bone marrow level in normal donors (white bar), in AS trait carrier donors (dashed bar), in SCA patients at baseline (gray bar), and after 60 days post-transplant (black bar). (B, C) Flow cytometric analysis of erythroid compartment for caspase 3 expression in SCA patients at baseline (B) and after 60 days post-transplant (C).

An initial increase in caspase 3 was observed after HSCT, as has been reported for later steps of “normal” erythroid cell maturation. Average percentages of caspase 3+CD71+CD45− were 1.8 ± 1.8% in normal AA donors, 2.7 ± 2.2% in AS trait carriers donors, 2 ± 1.5% in SCA patients at baseline, and 3.7 ± 4.3% in SCA patients at 60 days after transplant (Fig. 5).

## Discussion

The morbidity and mortality associated with SCA are much more frequent and severe than those associated with thalassemia. The Black African SCA variant manifests a severe phenotype when compared to the non-Black African SCA. It is possible that in patients with Black African SCA, a hyperplasia of the erythroid lineage exists at baseline, probably to maintain a constant production of erythroid precursors. The high level of polymerization of the sickle hemoglobin in host RBCs, as well as in host early and basophilic normoblasts, might also determine mechanical defects that, in turn, increase host cell susceptibility to clearance and loss.

The presence of ineffective erythropoiesis in SCA is supported by previous studies, which have identified structural abnormalities in SS erythroid precursor cells, thus indirectly indicating the increased susceptibility of these cells to clearance and loss. Blouin *et al.* examined erythropoiesis in the SCA mouse model and found significant morphological alteration in erythroid lineage late precursors (polychromatophilic normoblasts) within the marrow.^20^ These morphological studies identified a high level of hemoglobin polymers that were associated with increased cell fragmentation occurring during medullary transendothelial migration of reticulocytes. Older ultrastructural studies of BM aspirates derived from SCA patients identified reticulocytes that contain bundles of hemoglobin S polymers in the absence of intentional deoxygenation, as well as sickling of nucleated erythroblasts and extensive marrow erythrophagocytosis.^21^,^22^

Hasegawa *et al.* also found in an in vitro system that cultured nucleated erythroid precursors can undergo sickling under deoxygenating conditions 23. Recently, our group observed the presence of sickled erythrocytes at the BM level in SCA patients, in the absence of systemic symptoms, as well in AS trait carriers. This condition could be induced by the cellular stress of the biopsy procedure or alternatively represents a specific status of the BM of SCA patients, both the homozygous (SS) and heterozygous (AS) status (manuscript in preparation).

Apoptosis is an important mechanism by which ineffective erythroblasts are cleared within the intramedullary space, and our data suggest that Fas might contribute to the cell death of host erythroid precursors in SCA. If accelerated apoptosis is not compensated by enhanced erythropoiesis, however, clinically relevant anemia develops. Our studies suggest that significant abnormalities in SS erythroid precursors exist within the intramedullary space and that cells prone to sickling may be selectively destroyed prior to release from the erythropoietic compartment.

With HSCT, it is possible to give more than 90% chance of cure for children with SCA, with excellent survival rate and return to normal life. We agree with the recommendations of the Haematologica’ s authors.^25^ The young patients with symptomatic SCA, who have an HLA-matched sibling donor, should be transplanted as early as possible before sickling complications appear. The vast majority of our patients are not regularly transfused/chelated, or highly sensitized due to receiving RBC transfusions without the use of leukodepletion filters. Despite the recognized benefits of transfusion therapy, it is not without the risks of iron overload, alloimmunization, and delayed hemolytic transfusion reactions. Alloimmunization to RBC antigens is a major complication associated with RBC transfusions in patients with SCA. Alloantibodies and autoantibodies complicate RBC cross-matching, delay provision of transfusions, and increase the labor and cost of providing compatible RBC units. For these reasons and since patients had an HLA-identical sibling donor, these patients had indications for hematopoietic stem cell transplant.

After HSCT, we observed a “normalization” of erythroid populations, in parallel with a decreased intramedullary apoptosis rate, suggesting normal erythroid maturation in ex-SCA patients. In fact in the basal state, the erythropoietic system continuously produces excess numbers of early erythroblast, which become apoptotic through Fas-mediated signaling. The principal advantage of a homeostatic mechanism that relies on negative autoregulation of cell numbers is that it would self-correct for small perturbations, maintaining a relatively constant erythroblast population size in the basal state. The major expression of Fas at the early stage of erythroblast maturation has been observed at baseline in our patients, contributing to a negative autoregulation of cell number. After transplant, a tendency to a normalization of erythropoiesis has been observed in our patients, with a reduction of Fas expression on three erythroid population but especially on more mature erythroid precursors Ery C. A progressive maturation advantage for homozygous hemoglobin A (AA) or heterozygous hemoglobin S/hemoglobin A (SA) donor erythroid precursor cells resulted in a greater donor contribution to overall erythropoiesis following stem cell transplantation and improvement of clinical manifestations.

## Conclusions

This study suggests a good chance of cure for children with SCA, with HLA-identical transplant. We also observed “normalization” of erythroid populations in parallel with a decreased intramedullary apoptosis rate, suggesting normal erythroid maturation in ex-SCA patients after HSCT. HSCT should be considered the standard of care for SCA children with human leukocyte antigen-identical donor before complications result from the sickling of red blood cells.

## Figures and Tables

**Table 1:** Characteristics of patients (all Nigerian) harboring the Black African variant of SCA

UPN.	Age/Sex	HbS (%)	HbF (%)	Donor HbS (%)	Frequent VOC	Acute chest syndrome	Arterial ischemic stroke	No. Tx
166	14/M	76	22	30	yes	yes	Yes	14
191	6/F	70	27	41	yes	yes	No	0
192	9F	75	28	37	yes	yes	No	0
195	11/F	71	27	0	yes	yes	Yes	3
199	15/F	86	10	36	yes	yes	no	3
201	17/M	89	7	0	yes	no	no	0
202	3/M	85	11	35	yes	no	no	1
204	12/M	92	3	0	yes	no	no	0
205	5/M	79	17	0	yes	no	no	0
206	7/F	83	14	0	yes	no	no	0
207	13/M	88	8	41	yes	yes	no	3
210	3/M	73	24	37	yes	no	yes	0
213	16/M	91	5	0	yes	no	no	0
215	2/M	87	10	40	no	no	no	0
216	14/F	92	4	0	yes	yes	no	2
219	15/M	91	5	28	yes	no	no	15
221	4/F	83	15	31	yes	yes	no	0
222	16/M	91	5	0	yes	no	no	1
224	5/F	61	3	35.8	yes	yes	no	0
229	17/M	82	14	0	yes	yes	no	2
231	5/M	88	7.4	37	no	no	yes	0
234	15/F	94	1.9	42.1	yes	yes	no	2
235	8/F	77	na	0	no	no	yes	2
236	17/M	63	9.8	0	no	no	no	2
237	10/F	85	12.3	41.3	yes	no	no	0
239	13/F	73	3.7	39.4	yes	no	no	1
240	12/F	80	16.9	42.5	yes	no	no	1

HbS, hemoglobin S; HbF, hemoglobin F; VOC, vaso-occlusive crisis; No. Tx, number of red blood cell transfusions received before transplant; na, not available.
